# Evidence of forest restoration success and the conservation value of community-owned forests in Southwest China using dung beetles as indicators

**DOI:** 10.1371/journal.pone.0204764

**Published:** 2018-11-08

**Authors:** Casey D. Sullivan, Eleanor M. Slade, Ming Bai, Kun Shi, Philip Riordan

**Affiliations:** 1 The Wildlife Institute at Beijing Forestry University, School of Nature Conservation, Beijing Forestry University, Haidian District, Beijing, China; 2 Lancaster Environment Centre, Lancaster University, Lancaster, United Kingdom; 3 Department of Zoology, University of Oxford, Oxford, United Kingdom; 4 Key Laboratory of Zoological Systematics and Evolution, Chinese Academy of Sciences (CAS), Institute of Zoology, Chinese Academy of Sciences, Chaoyang District, Beijing, China; 5 Eco-Bridge Continental, Huizhi Tower, Haidian District, Beijing, China; 6 Marwell Wildlife, Thompsons Lane, Hampshire, United Kingdom; Assam University, INDIA

## Abstract

Protection of the world’s remaining forests and biodiversity is a matter of global concern. Yunnan, China is home to China’s only mainland tropical rainforests, and 20% of China’s total biodiversity. Despite restoration measures and establishment of new protected areas, this region is still experiencing biodiversity loss due to inadequate management and monitoring. We evaluate restoration success of China’s tropical forests in Xishuangbanna National Nature Reserve (XSBN-NNR), Yunnan, China using dung beetles as an indicator taxon. We sampled across a land-use gradient of human alteration: protected forest, restored forest, community owned forest, and rubber plantation. We collected 3,748 dung beetles from 21 species over a 3 month period. Multivariate analyses revealed unique assemblages in each land-use category, but with restored forest most similar to protected areas, suggesting restoration success in this region. Community forests were more diverse than plantations, suggesting that community forests may be a valuable and practical conservation tool in this region. Most species were generalists, although some had dietary and habitat preferences. Furthermore, dietary niche breadths were, on average, higher in disturbed areas, suggesting that disturbance may result in dietary changes. We show that restoration of tropical forests appears to be successful for a key ecological and biological indicator group- dung beetles. Furthermore, community-owned forests appear to be valuable and practical method of maintaining ecosystem health and biodiversity in the region. Future management in this region would likely benefit from encouragement to maintain community-owned forests, economic incentives for restoring farmland to forest, and increased environmental monitoring across the land-use gradient.

## 1.1: Introduction

Protecting the world’s remaining biodiversity, particularly in tropical regions, is a priority [1–3]. Over the last two decades, global tropical deforestation has exceeded an average of 12 million hectares annually [4]. This loss has been particularly acute in South-East Asia, a recognized biodiversity hotspot [5]. Restoration efforts are increasingly being used to combat this loss, with some regions even increasing in forest cover [6]. While restoration may contribute to biodiversity gains, this relationship is poorly understood, and some studies suggest that greater intervention may be necessary to restore biodiversity and prevent irrevocable loss of ecosystem functions [7–10]. In order to allocate funds and effort more effectively, it is important to monitor the biodiversity and health of remaining forests as well as evaluate the success of restoration efforts. Indicator taxa are increasingly used as a tool for monitoring ecosystem health and restoration success, especially in highly diverse tropical regions [11–13].

Indicator taxa can be defined as a species or an assemblage of species that have adapted to particular features of the ecosystem and reacts predictably and rapidly to change [14]. Dung beetles (Coleoptera:Scarabaeidae) are examples of potentially good indicators, given their inherently close relationship with both flora and fauna, particularly mammals [11,15,16], and their responses to environmental change [17,18]. Their relative ease of sampling [19], high species turnover rate (β diversity), global diversity, and well-characterized taxonomy [11,16] offer additional advantages.

Dung beetles provide a variety of ecosystem services, including nutrient cycling, soil aeration, and seed dispersal [20]. Widespread losses of dung beetles potentially results in degradation of ecosystem functioning [20–22]. Thus, dung beetles can be used to indicate the success of restoration activities for biodiversity and offer evidence for the restoration of ecosystem functioning [7].

Examples of the use of dung beetles as indicators in tropical and sub-tropical south-east Asia include assessment of forest fragmentation following timber extraction, and palm oil and rubber plantation development (i.e. [23–26]). Here we use dung beetles to examine forest restoration in the poorly studied tropical forests of southern China.

Yunnan Province, bordered by Myanmar (Burma), Laos, and Vietnam, contains some of China’s last remaining tropical rainforests. It lies within the Indo-Chinese biodiversity hotspot [27], and is also one of only two regions in China where rubber (*Hevea brasiliensis* (Willd ex. A.Juss.) Müll. Arg.) can be cultivated. Since the 1970s, rubber plantations have increasingly become a major land-use category in Yunnan due to the crop’s economic potential [28–31]. This is particularly apparent in Xishuangbanna National Nature Reserve (XSBN-NNR) in southern Yunnan. Currently, rubber plantations cover 10% of XSBN’s total area [32] and less than 50% of the original natural forest remains [33]. XSBN is China’s last remaining safe-haven for Asian elephants (*Elephas maximus* Linnaeus, 1758), and contains 22% of China’s vertebrate diversity, despite only covering 0.02% of China’s land area [34,35]. XSBN has also experienced deforestation in the form of replacing traditional agriculture with non-rubber commercial plantations, such as fruit, and infrastructure development [28,33,36,37]. Government policies for forest restoration have been underway since 1999 as part of the “Restoring Farmland to Forest” or “Grain for Green” (退耕还林) action [38], however, the success of these policies in XSBN has not been fully evaluated. Surveys of birds [39] and plants [40] have underlined the need to improve protected area management practices and restoration measures, but effective monitoring has been highlighted as a priority [41,42]. Presently, despite the conservation value of Xishuangbanna, there is limited funding and capacity for long-term ecological monitoring [35,43,44]. Thus, monitoring efforts must be simple and affordable if they are to be effectively implemented. Dung beetles are an ideal group for this, allowing rapid sampling and assessment of ecosystem changes and health at minimal cost and labor [45].

We used dung beetles as an indicator of ecosystem health and restoration success across a human-modified gradient in the Mengyang sub-reserve of the XSBN-NNR system. We evaluated the diversity and trophic requirements of dung beetle assemblages across protected forests, restored forest, community-owned forests, and rubber plantations and explored these trends in terms of tropical forest ecosystem health in China. We then identified potential indicator species, which can be further tested and potentially used to monitor ecosystem change in the region in the future.

## 2.1: Methods and materials

### 2.1.1: Study area

This work was carried out in Mengyang reserve, located within the Xishuangbanna National Nature Reserve (XSBN-NNR) in the Dai Autonomous Prefecture of Xishuangbanna (XSBN), Yunnan. XSBN receives 1200mm-2500mm of rain annually, with 80% falling during the rainy season (May-October) [46,47] In addition to rainfall, heavy fog deposits water in the dry season and is credited with increasing the northern limits of tropical rainforests in this region [48]. This region contains four major natural vegetation types: tropical montane rainforest, evergreen broadleaf forest, tropical seasonal rainforest, and monsoon forest over limestone. XSBN is also home to over 1.1 million people from a diverse range of ethnic groups [49]. The XSBN-NNR system was established in 1958 and contains six reserves that cover a land area of approximately 2,500 km^2^. Of these reserves, Mengyang NNR is the largest, with an area of around 1,000 km^2^, with records of 283 species of birds, and numerous plant species [50]. The 59 recorded mammal species include Asian elephant, Indo-Chinese tiger *(Panthera tigris* Linnaeus, 1758), clouded leopard (*Neofelis nebulosa* Griffith, 1821), and several primate species [50].

**Fig 1:**
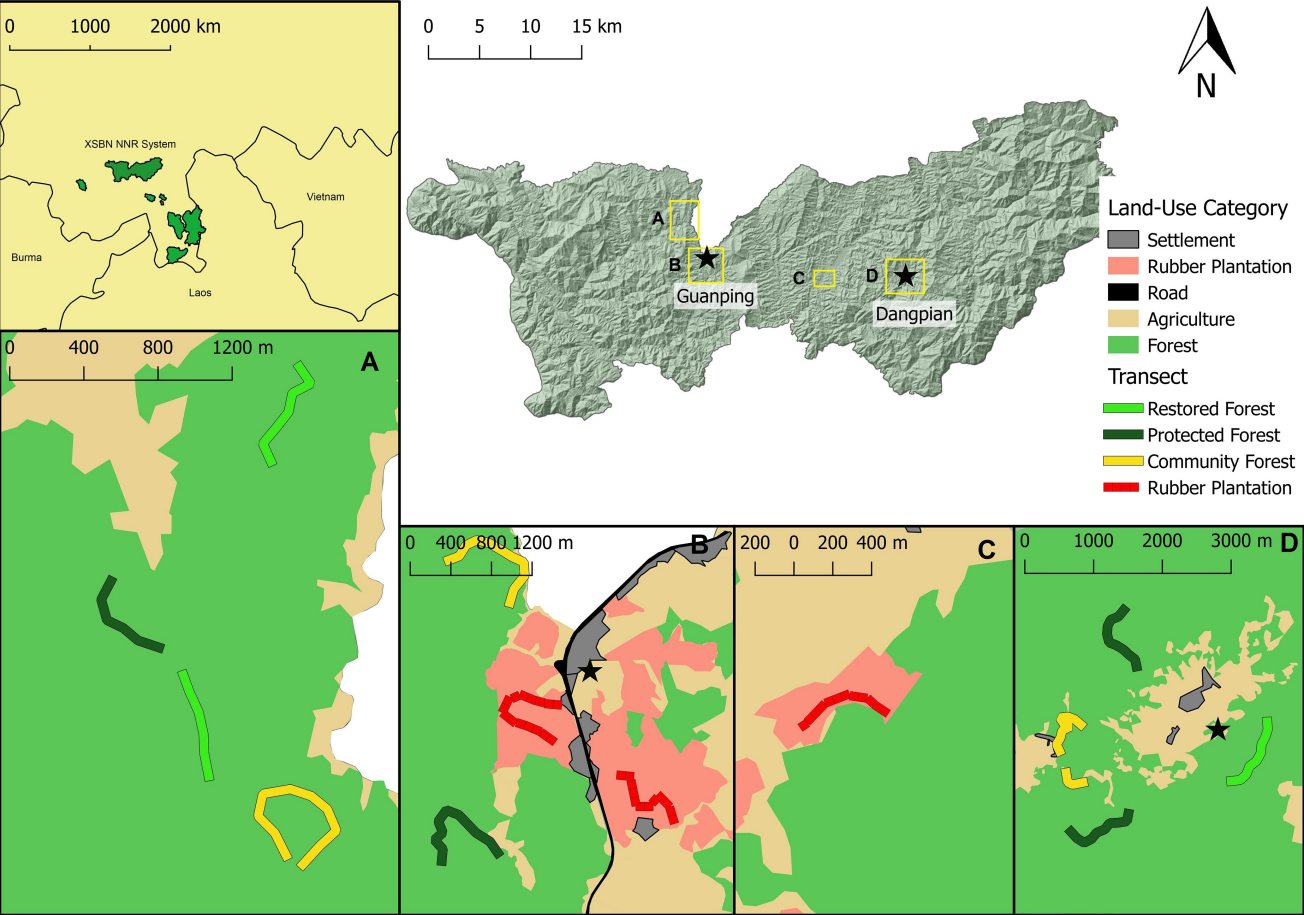
Location of XSBN-NNR and study sites. Location of XSBN-NNR within China and the position of study sites (Guanping and Dangpian, indicated with stars) and transects, with land-use categories in the vicinity of sampling locations. Land-use classification was developed using ArcGIS satellite imagery and Vegetation Continuous Fields (VCF) [52] with a forest cover cut-off of 70% to delimit mature rubber trees from forest [53]. Land used for corn, rice, substinence farming, and tea is classified as agriculture.

Two villages, Guanping (关平) and Dangpian (挡片), were chosen as foci for surveys in Mengyang NNR. Sampling was conducted within 22°17’25.8” and 22°14’18.96” N latitude; 100°52’24.49” and 101°3’40.64” E Longitude. Transects were positioned using habitat categories and local knowledge across a human-modified land-use gradient: rubber plantation (n = 3), community-owned forest (n = 4), restored forest (n = 3), and protected forest (n = 4) (Fig 1). Restored forests were defined as having previously been converted from farmland or plantation in or before 1999 under the “Restoring Farmland to Forest” or “Grain for Green” national forest restoration policy [51], which paid farmers to plant and encourage forest on marginal farmland. Community forests were categorized as forests belonging to a household or village where gathering and some timber extraction are permitted. All forests sampled had experienced some level of human disturbance. For example, we encountered evidence of poaching snares, harvested trees, and human refuse in even the most remote protected areas sampled.

### 2.1.2: Sampling

Dung beetle sampling using pitfall trapping was carried out once per month over three months during the rainy season (Aug–Oct, 2016). We sampled in the rainy season because dung beetle diversity is often higher during times of precipitation [54,55]. We used 1.8L plastic traps with dimensions of 18x12x11cm. Pitfall traps were set in groups of three (triplets) placed at least 7m apart, each of which had a different bait: carrion, omnivore dung, or elephant dung. Carrion consisted of pig meat left to decay for at least 6 hours, locally collected pig dung was used as omnivore dung, and elephant dung was collected from forest-grazed rescue elephants living in Mengyang’s “Wild Elephant Valley”. Seven meter separation between bait types has been used in other multiple bait studies aimed at capturing maximum diversity of dung beetles as well as diet specialists (e.g. [7,56–58]). It is acknowledged that this separation may not allow independence between baits [59], however, this method enabled a broader assessment of the full dung beetle community compared to using only a single bait type.

Triplets were placed at least 100m apart to ensure independence between trapping points [59] along transects of between 500m to 1km in length, depending on logistic accessibility and land-use section size. Transects started at least 100m from forest edges to minimize forest-edge effects, and at least 500m from paved roads. A total of 1,107 traps were set over the three sampling months: 333 were in protected forests, 243 in restored forests, 306 in community forests, and 225 in rubber plantations. However, baits were removed by wildlife from 12 traps in protected forests, 6 traps in restored forests, and one trap in both community and rubber plantations. These traps were removed from analyses, resulting in a final sampling effort of 1,087 traps.

We used 100g of homogenized bait in each trap to avoid potential size bias. Baits were wrapped in muslin fabric, which was then suspended across the top of the trap and secured on either side. Trap covers were placed approximately 10 cm above the trap to prevent depredation of beetles by birds or removal of baits by animals, as well as to limit rainfall entering the trap. In accordance with reserve permit requirements, traps were left dry and empty to minimize fatalities. Our pilot studies indicated that dung beetles could not crawl out of the traps or easily fly through the gap between the lid and the trap. However, we acknowledge that some specimens may have escaped or been depredated, for example by predatory beetles, such a staphalinids, entering the traps.

Trap contents were collected after 24 hours. In accordance with permit requirements, large and easily identifiable specimens were released after identification in the field. All small individuals, and specimens which could not be identified in the field, as well as representative specimens from larger species, were euthanized using a 5% chloral hydrate solution, followed by storage in 70% ethanol in the field and thereafter in 95% ethanol.

### 2.1.3: Data analysis

Collected specimens were identified to species level using museum specimens, and where this was not possible a morphospecies code was assigned. Specimens that were too damaged to be identified were excluded from further analyses (n = 7). Reference specimens were deposited in the Beijing Forestry University Museum. EstimateS [60] was used to calculate species representivity using four common richness estimators (ICE, ACE, Chao1, and Jackknife) and create species accumulation curves using 100 randomizations. All other data were analyzed using R statistical package, version 3.1.2 [61] and packages vegan [62], labdsv [63], iNEXT [64,65], MASS [66], lme4 [67], and LmerTest [68].

We first assessed diversity metrics across land-use categories and across bait types. To evaluate diversity metrics across land-use categories all individuals found at each bait type were summed for each triplet of traps (grids). Biodiversity metrics (Shannon’s H, Simpson’s D, Fisher’s Alpha, and abundance) were calculated for each grid. Linear mixed-effects models were used to assess the influence of land-use category on derived metrics followed by lsmeans pairwise comparison. As this was a repeated measures survey with sampling conducted over several months we used a random intercept for sampling month, and grids nested within transects to deal with spatial pseudo-replication [69]. Abundance values were log-transformed prior to modeling. Rarefied species richness was calculated for the entire transect using coverage-based rarefaction methods in iNEXT [64]. The models for rarefied richness were constructed with a quasipoisson error distribution using glmmPQL in MASS [66] with transect nested within land-use type.

We repeated the above process to analyze diversity metrics across bait-types, with each individual pitfall trap representing an individual data point. Again, a random intercept was used for sampling month with grids nested within transects, and GPS points for each sample nested within grids. Rarefied richness was calculated at the transect level for each bait-type, and abundance values were log-transformed prior to modeling.

We used a distance matrix based multivariate permutational analysis to test for differences in dung beetle assemblages across land-use categories and bait types. Firstly, we assessed the variance in assemblages explained by land-use categories, and then assessed the variance explained by bait types, taking into account the variance explained by land-use category. Grouping factors were used to constrain permutations with respect to individual transects and the month. We used the metaMDS function in the Vegan package to determine the optimum Bray-Curtis dissimilarity matrices for the abundance matrices, which resulted in Wisconsin standardization. We then used non-metric multi-dimensional scaling (NMDS) to map species assemblages across land-use categories and bait types.

The potential of each species as an indicator species was tested using the indicator valuation method, IndVal [70], with 1000 permutations to evaluate habitat specificity and fidelity among dung beetle species. IndVal scores are a product of the species relative frequency and relative abundance, expressed as a value between zero and one [62,70]. Indicator species for specific land-use categories were evaluated against a threshold value of ≥0.70, with species scoring below this threshold regarded as generalist species [13,71].

Finally, we calculated Levin’s Niche Breadth (Levin’s D) for each species in each land-use category in order to evaluate the relationship between species’ abundances and dietary niche breadths (e.g. [72,73]).

Individuals of each species were summed within transects with respect to bait type, and Levin’s values (D) were determined for each monthly sampling repetition. Due to some species not being present in a given transect, a total of 153 values were calculated. We then calculated abundance for each species and total abundance of all species, observed richness, and coverage based rarefied richness for each transect in each monthly repetition. We used linear mixed effects models to evaluate the relationship between Levin’s D and the abundance of each species, total abundance of all species, observed species richness, and rarefied species richness to determine whether niches were broader in depauperate habitats and for more abundant species.

We first created the global model in lme4 with all variables (land-use category, species identity, rarefied species richness, total abundance at each transect, and each species’ abundance at each transect, and logical two-way interactions). Two-way interaction variables between land-use-category and total abundance of all species, the abundance of each species, and species richness were modelled, in addition to the interaction between each species and its abundance. Abundance values were log-transformed prior to analysis. The Step function from lmerTest [68] was then used to simplify the global model by removing non-significant effects. The random effects structure used random intercepts for sampling month and nested transects within land-use category.

## 3.1: Results

### 3.1.1: Richness estimations and sampling representivity

In total 3,741 individual dung beetles were identified, representing 21 species or morphospecies across seven genera. We identified 1,407 individuals from protected forests (19 species), 1,028 individuals from restored forests (19 species), 1,056 individuals from community forests (18 species), and 250 individuals (14 species) from rubber plantations (S1 Table). Species accumulation curves approached asymptotic levels for all land use types (S1 Fig). There was an indication that ACE, ICE, Chao 1, and Jackknife means came close to convergence for community forests and rubber plantations, but ICE and Jackknife appeared to continue increasing in restored and protected forests (S2 Fig).

### 3.1.2: Biodiversity metrics and community assemblages across land-use categories

**Fig 2:**
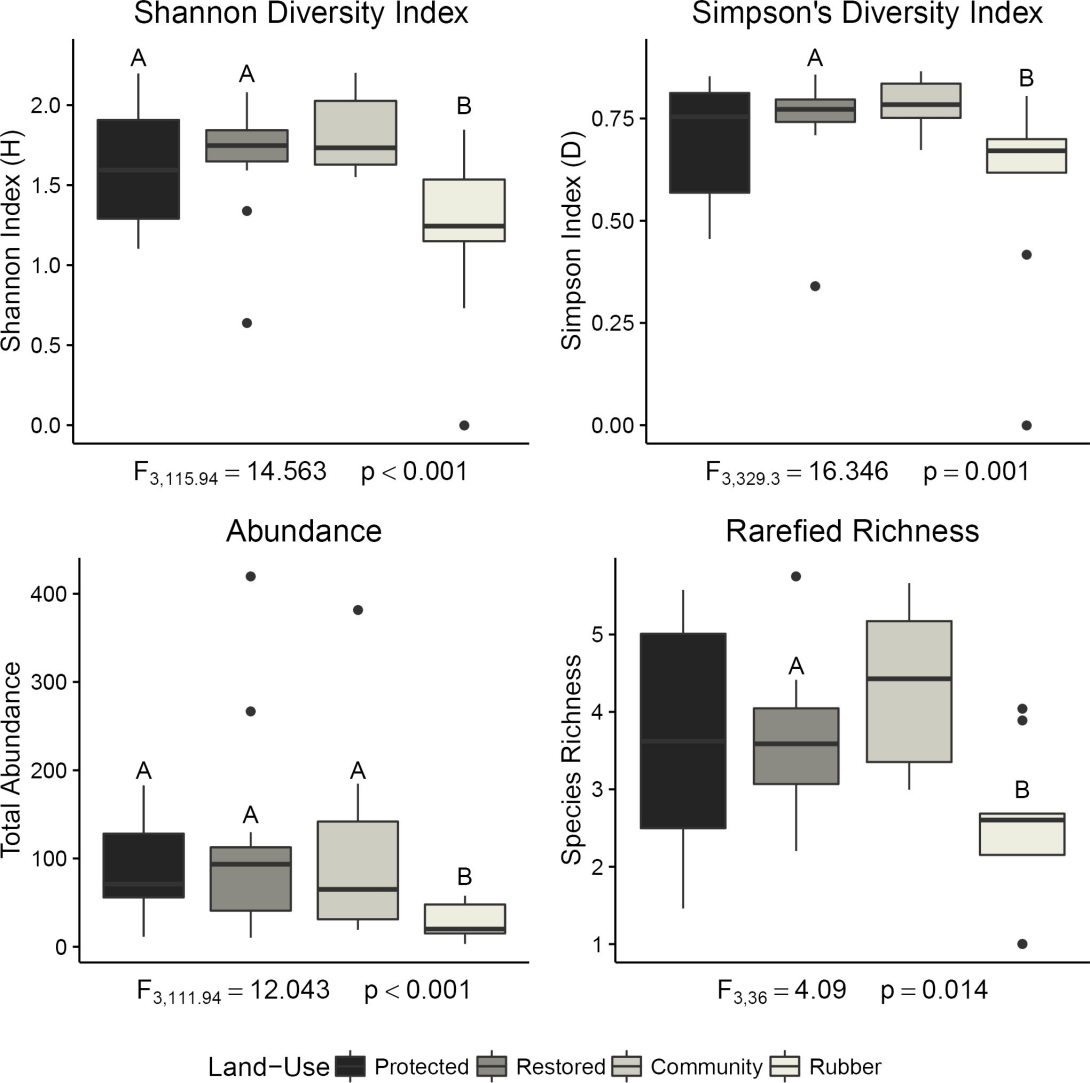
Diversity metrics for dung beetle assemblages across land-use categories. Diversity metrics, excluding Fisher’s Alpha (which was not significant), across land-use categories at the transect level. Transect means are shown, along with GLM model statistics against the null model. Letters above the error bars indicate significant differences (p<0.05) between land-use categories, as determined by pairwise lsmeans. Denominator degrees of freedom using Satterthwaite approximation are shown.

All of the models for biodiversity metrics were significant against the null model except for alpha diversity (F_3, 117.71_ = 1.43, p = 0.238) (Fig 2). None of the forested land-use categories (protected, restored, and community-owned) differed from each other across any of the biodiversity metrics. Rubber had significantly lower diversity metrics than restored forest for all significant models, but was only significantly lower than protected and community forests for Shannon’s H and abundance (Fig 2). Rarefied richness was significantly lower in rubber plantations than in restored forests, but not protected forests or community forests. Fisher’s alpha was not significantly different across any land-use category.

**Fig 3:**
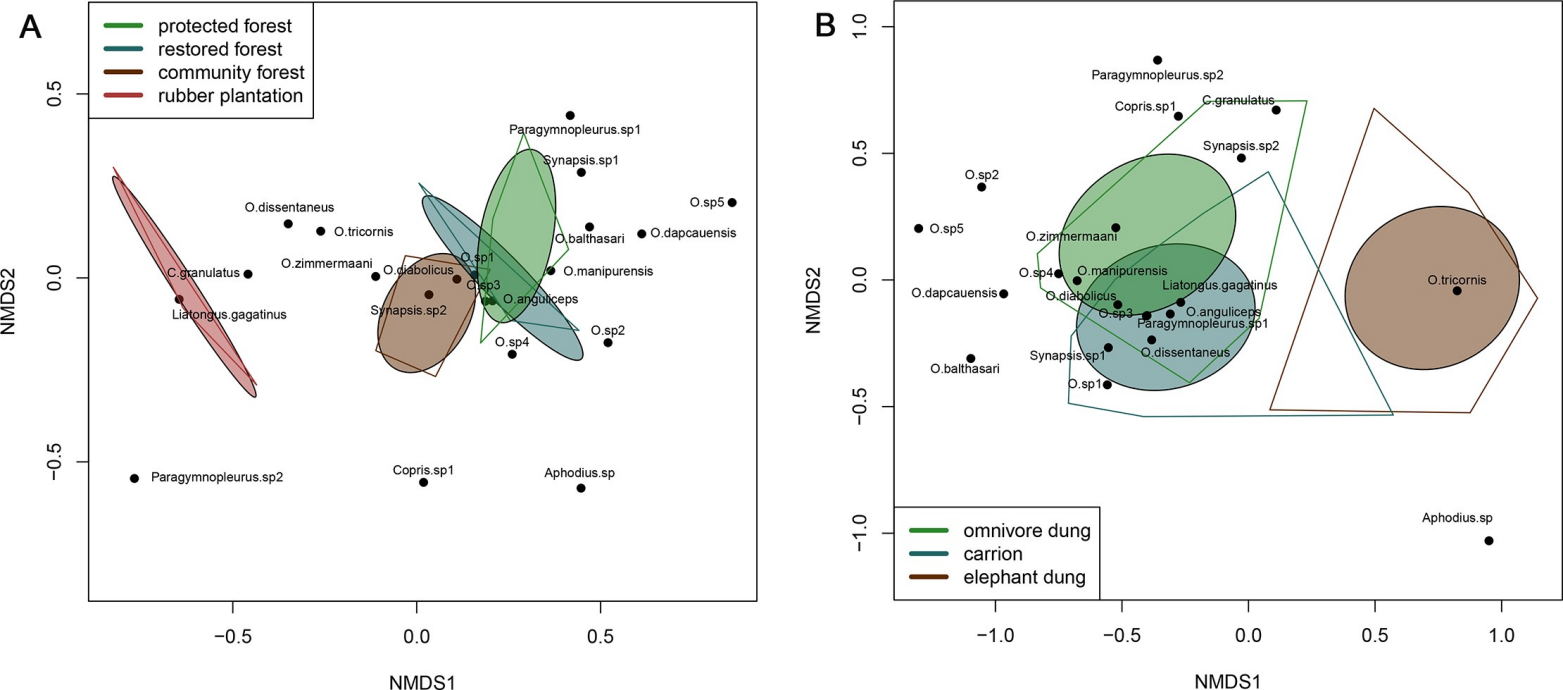
NMDS ordinations of dung beetle assemblages across land-use and bait type categories. NMDS ordinations for assemblages across A) land-use categories (stress = 0.11) and B) bait types (stress = 0.17) using Wisconsin standardization of abundance matrices. Ordinations include standard deviation ellipses (CI = 0.05) and convex hulls enclosing all data points within each grouping level (land-use category, bait type, respectively).

Dung beetle community assemblages did not vary significantly within transects, between transects of the same land-use category, or between sampling months (p>0.05). However, there was significant variation in dung beetle assemblages across land-use categories (F_3, 41_ = 2.42, p<0.01). NMDS ordinations showed complete separation of 95% confidence interval ordination ellipses for rubber plantations and the forested land-use categories (Fig 3A). However, the three forested land-use categories were closely clustered, and protected forests and restored forest assemblages overlapped. Convex ordination hulls, which enclose all points within each land-use category, were consistent with the 95% confidence ellipses.

### 3.1.3: Land-use indicator analysis

We identified 10 species with significant indicator scores. Significant indicators were identified for all forest types except for community forest (Table 1). The majority of indicator species were for restored forest, with the highest indicator values of 0.38 for *Onthophagus zimmermaani* and *O*. *diabolicus*. Only one indicator was identified for rubber plantations, *Liatongus gagatinus*, and protected forests, *Onthophagus anguliceps*.

**Table 1:** Abundance of species with significant indicator values.

	Community Forest	Protected Forest	Restored Forest	Rubber Plantation	Indicator Value	Cluster
*Liatongus gagatinus*	5	2	6	17	0.15**	Rubber
*Onthophagus anguliceps*	138	377	162	46	0.30**	Protected
*O*. *balthasari*	0	1	9	0	0.15***	Restored
*O*. *diabolicus*	99	150	178	14	0.38***	Restored
*O*. *dissentaneus*	31	41	33	26	0.17*	Restored
*O*. *manipurensis*	35	72	85	1	0.27**	Restored
*O*. *zimmermaani*	23	17	76	9	0.38***	Restored
*O*. *sp3*	174	299	165	38	0.29*	Restored
*O*. *sp4*	14	10	37	1	0.13***	Restored
*Synapsis sp1*	8	29	14	0	0.17**	Restored

Abundances of species with significant indicator values in each land-use category, their indicator values, and their associated cluster. Only species with significant indicator values are shown. Levels of significance

<0.001***

<0.01**

<0.05*.

### 3.1.4 Variation in biodiversity metrics and assemblages across bait types

We collected 1,780 individuals from omnivore baited traps, representing 48% of total individuals captured. Carrion baited traps were similar, with a total of 1,695 individuals, or 45% of total individuals. Elephant dung collected 266 individuals, representing only 7% of the total number of individuals captured. Total observed species richness was highest for omnivore dung (n = 20), followed by carrion (n = 17), and finally elephant dung (n = 14) (S2 Table).

Permutational analysis revealed that dung beetle assemblages also varied significantly between bait types, explaining 19% of the observed differences in assemblages, after accounting for variations between land-use categories (F_2, 117_ = 14.11, p<0.001). In the ordination, the 95% confidence ellipses for omnivore and carrion baits overlapped significantly, but elephant dung assemblages were entirely non-overlapping. However, the convex hull for carrion baits did overlap the other two bait types (Fig 3B). The assemblage of dung beetles captured in each bait type appeared to be a subset of the overall assemblage, although *Aphodius sp*. (n = 2) was only found on the elephant dung bait.

**Fig 4:**
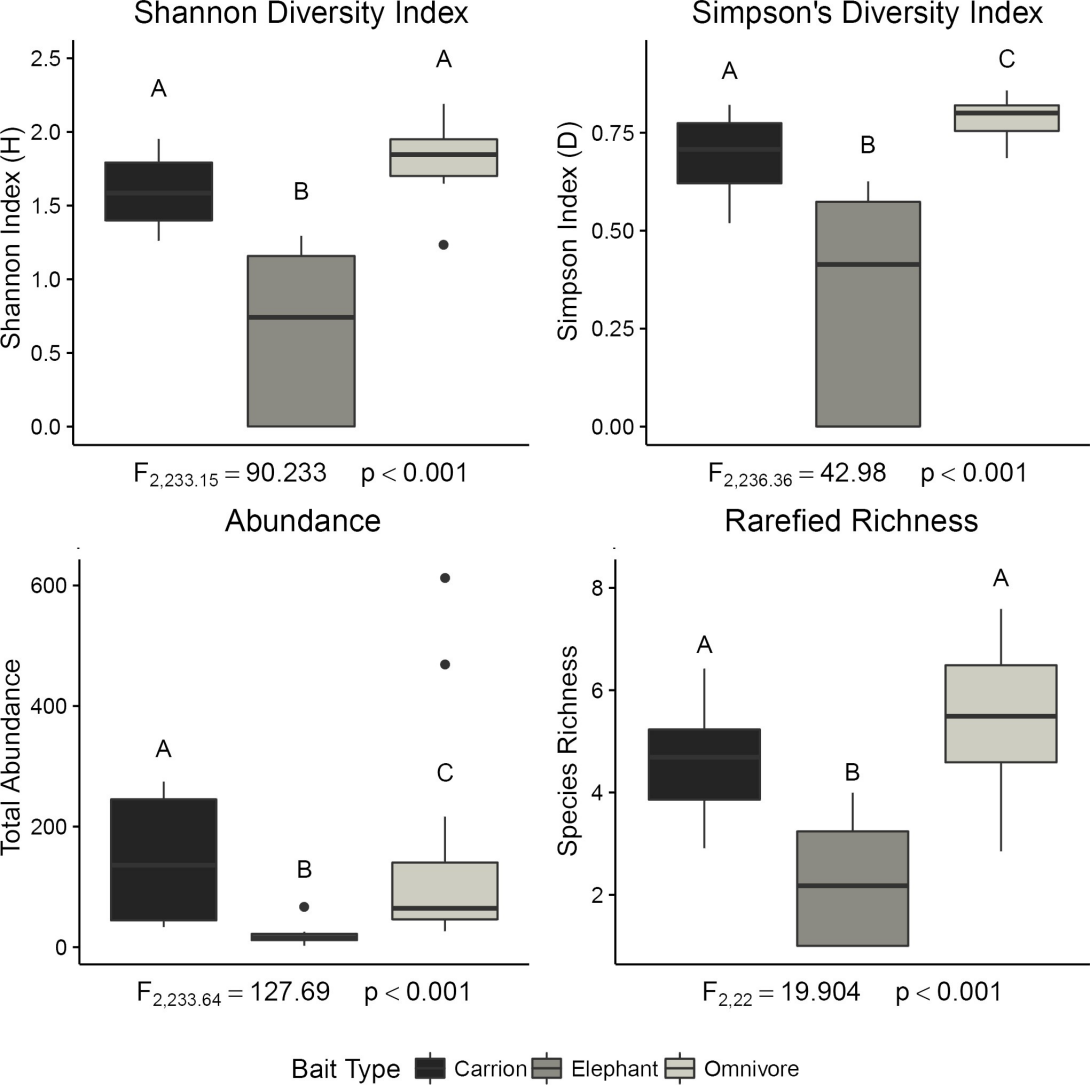
Biodiversity metrics of dung beetle assemblages across bait types. Transect means for each biodiversity metric at the transect level with standard errors. Letters above the standard error bars indicate significant differences (p<0.05) between bait-types, as determined by lsmeans pairwise comparisons.

Simpson’s D, Shannon’s H, log transformed abundance, and rarefied richness were all significant compared to the null models. Diversity values were significantly lower for elephant dung than for the other two bait types across all models. Carrion values were significantly lower than omnivore in the model for log-transformed abundance and significantly higher than omnivore baits in the models for Simpson’s D (Fig 4).

### 3.1.5: Variations in niche breadth across land-use categories

**Fig 5:**
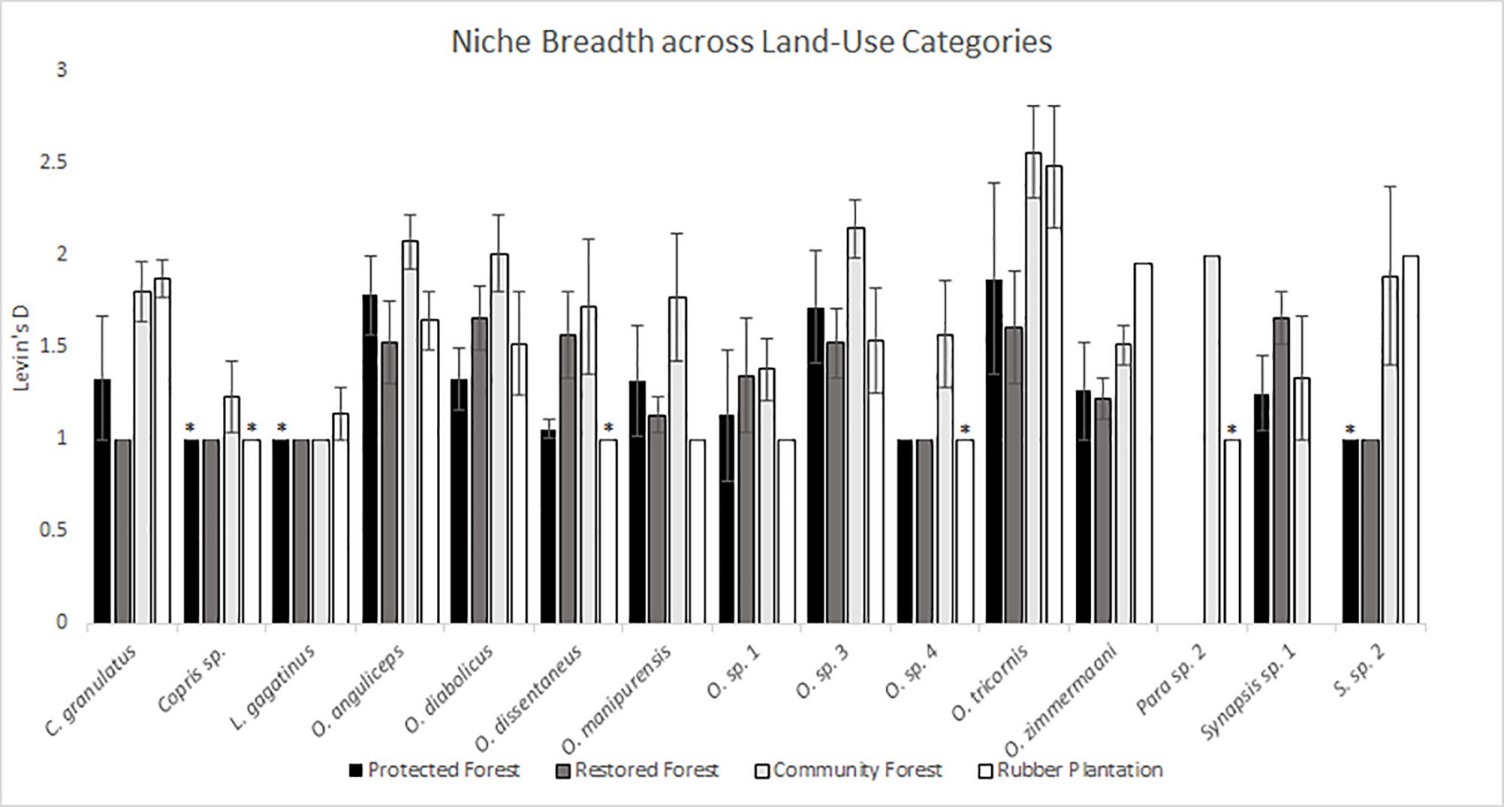
Comparison of Levin’s niche breadth for species across land-use categories. Averaged Levin’s niche breadth values for each species at each site type with standard errors. Species found on only one bait type (D = 1) are omitted (*Paragymnopleurus sp*. *1*, *O*. *balthasari*, *O*. *dapcauensus*, *O*. *sp*. *2*, *O*. *sp*. *5*, *Aphodius sp)*. Species without standard error bars were either only present during one sampling period (as indicated with an asterisk) or experienced no variation in niche breadth.

Levins’ Niche Breath Values (D) for most species were greater than 1, but several species (*Paragymnopleurus sp*. *1*, *O*. *balthasari*, *O*. *dapcauensus*, *O*. *sp*. *2*, *O*. *sp*. *5*, *Aphodius sp*), had a niche breadth of 1, meaning they were found on only one bait type. On average, niche breadth was higher in disturbed areas—community forest and rubber plantations, than in undisturbed areas—protected forest and restored forest (Fig 5).

The final, simplified model for Levin’s Niche Breadth included only co-variates for species, species’ abundances, the land-use category, and the interaction between species and their abundances. Rarefied richness, total transect abundances, and interactions between these variables were all removed during the model simplification process (S3 Table).

Least square (LS) means showed a similar trend to standard mean values for niche breadth across land-use categories: more highly disturbed categories (community forest and rubber plantation) had higher values (1.39±0.07, 1.38±0.08, respectively) than less disturbed categories (protected forest, restored forest; 1.22±0.07, 1.22±0.07, respectively). LS mean values were significantly higher in community forests than protected and restored forests (p = 0.02, both), but differences between community forest and rubber plantations were not significant (p = 0.89). While rubber plantations had a higher mean value than restored and protected forests, this difference was not significant (p = 0.06, both).

The LS mean values were significant at the p<0.05 value for all species except *O*. *dapacauensis* (p = 0.05). Several congeneric species differed significantly, including *O*. *tricornis*, *O*. *zimmermaani*, and *O*. *dissentaneus* (S4 Table). Despite significant variation between species’ niche breadths, and significant variations in niche breath across land-use categories, the interaction variables between land-use categories and species derived values (abundances and richness) were not significant.

Species abundance, overall, had a significant positive relationship with niche breadth values (Estimate = 0.51±0.11, p<0.001). However, the species by species abundance interaction was negatively related to niche breadth, except for *Synapsis sp*. (Estimate = 0.002±0.28, p>0.05).

## 4.1: Discussion

Overall, our results are consistent with numerous other studies in SE Asia that show that dung beetle assemblage diversity decreases with increasing disturbance (e.g. [17,74–77]). Our results show that restoration of farmland to forest also restores dung beetle communities with restored forests showing increased diversity and abundance of dung beetles compared to plantations and community forests and community compositions most similar to protected forest. While community-owned forests had lower dung beetle diversity and abundance than restored forests, they may represent a practical tool for conservation by maintaining higher levels of diversity than plantations. Thus, these managed areas of forest may be more vital for ecosystem health at a landscape level than previously realized. Importantly, we show that dung beetles may act as reliable indicators of habitat disturbance and ecosystem health for the XSBN-NNR region, and could be used to monitor future land-use changes.

### 4.1.1: Changes in dung beetle communities across the land-use gradient

Relatively high dung beetle diversity in restored forests and community forests suggests that these habitats are capable of retaining or recovering biodiversity. Higher diversity and species richness in forested (protected, restored, community-owned) land-uses compared to unforested land-uses (rubber) is consistent with many other studies on dung beetles in SE Asia [24,25,74,77–80] and other groups, such as birds [26] and mammals [81]. However, the ability of restored forests to fully regain biodiversity is not well studied, although some studies have indicated that prolonged restoration efforts have not resulted in fully restored biodiversity [7,82,83] or have required considerable intervention and management [9,84]. The success of restoration activities in XSBN-NNR may be because the restored forests sampled are all on the edge of, or within, non-restored protected forests [85].

Rubber plantations had significantly lower richness compared to most forested areas, and lower total abundance. This is not surprising, and is consistent with findings regarding plantations from other studies in similar regions (e.g. [17,24,45,80,86]). Community forests were distinct from the other two forest types, but still more similar to protected and restored forests than to rubber plantations. This suggests that community forests represent a practical tool for local forest conservation, especially when compared to monocultures, while still allowing people living in the area to directly benefit from the forest. Few studies have looked directly at diversity within community-owned forests but a link between community management and higher biodiversity in this region has previously been noted [43]. Community forests in other regions of SE Asia have been credited with alleviating poverty, as well as increasing biodiversity and biomass production [87–89]. However, within this region, we observed community forests being replaced by plantations in some areas. We suggest that encouragement to maintain community forests instead of converting them into other land-use categories may have significant benefits for biodiversity as well as human wellbeing (e.g. [90–92]).

Despite distinct assemblages across land-use categories, no indicators met the specialist cut-off value of 0.70 [13,71]. However, many species showed a significant preference in land-use categories, suggesting that they are flexible generalists that may serve as “detector species”, or species that show habitat preferences, but will still move to adjacent habitats under changing environmental conditions [71]. Therefore, these species may be useful for monitoring further restoration success.

### 4.1.2: Dung beetle diet preferences

Almost all individuals (94%) were collected from omnivore and carrion baited traps. These baits captured similar total abundances, although omnivore dung had slightly higher observed richness. Elephant dung captured only 6% of the total individuals and only 14 species. While the attraction of smaller dung beetle species to elephant dung has been recorded elsewhere in Asia [93], we are not aware of any studies showing lack of attraction to elephant dung in areas where elephants are native and present. However, one limitation of our study was that we used standardized bait sizes of 100g, which is much smaller than the size of a typical elephant dung pat and may have influenced dung beetle assemblages. The use of captive elephant dung in this study may have influenced dung beetle responses, although the captive elephants used as a dung source in this study were grazed in the forest and exposed to a similar diet to the wild elephants. Furthermore, all wild elephant dung encountered during the study was inspected for dung beetles, but no new species were found, and the dung often contained low or zero individuals.

### 4.1.3: Variation in niche breadth

Levins’ niche breadth varied significantly across species, with congeneric species showing different bait preferences. Niche breadths were, on average, higher in community forests and rubber plantations than in less disturbed forests. However, the interaction terms for species-specific variables by land-use category were not significant, suggesting that individuals, as opposed to species as a whole, may adjust their niche breadth based on differences in their habitat in this study site. Additionally, total abundance of all dung beetles in a transect was not significant, although each species individual abundance was, suggesting that interspecific competition may have less influence on niche breadth than intraspecific competition.

Despite significant variation between bait types, we acknowledge that our traps were not necessarily independent of each other. The trap spacing of 7m, selected in accordance with other studies that aimed to maximize sampling representivity by using multiple bait types (e.g. [7,56–58]) may have resulted in interference among bait types, particularly with strong-smelling baits like pig dung and rotting meat. More research regarding interspecific dietary niche breadth is needed to determine whether the diet plasticity exhibited by dung beetles is due to evolutionary responses at the population level or individual behavioral responses.

## 5.1: Conclusion

This research provides a baseline for further studies on ecological health, conservation, and restoration success of forested tropical and sub-tropical areas in China. This region is of both conservation and economic interest, and protecting biodiversity while maintaining economic opportunities for the people living there is vital. We show that restoration of tropical forests appears to be successful for a key ecological and biological indicator group—dung beetles. Furthermore, community-owned forests appear to be a valuable and practical method of maintaining ecosystem health and biodiversity.

The low number of species with either dietary or habitat specialisms suggests a possible decline in specialists due to habitat change. Unfortunately, no previous dung beetle surveys exist for XSBN, making comparisons over time impossible. Therefore, it is difficult to determine if XSBN has reduced richness and a loss of specialists, or if this is just a natural consequence of biogeography. We encourage further work across latitude gradients to understand the distributions of dung beetle assemblages in SE Asia, as well as surveys in more isolated regions of the protected areas where mammal diversity remains relatively high and during both the rainy and dry season. Combining dung beetle surveys with extensive mammal surveys will provide a better understanding of interspecific interactions and trophic networks in this highly fragmented landscape.

As China continues to create and improve its protected areas, monitoring their success is increasingly vital. The presence of markedly different assemblages across land-use categories suggests that dung beetles should continue to be used as indicators of restoration and protected area success for this region. In addition, land-management in this area would likely benefit from maintenance of community-owned forests, and restoration of farmland to forest.

## Supporting information

S1 Table Species abundances across land-use category. Number of individuals of each species or morphospecies captured in each land-use category, and total species abundances and total abundances for each land-use category.
(DOCX)

S2 Table Species abundance across bait types. Number of individuals of each species or morphospecies on each bait type and total observed abundances and observed richness for each bait.
(DOCX)

S3 Table Levin’s niche breadth model statistics. Summary of the null model, global model, and final simplified models evaluating Levin’s Niche Breadth, with AIC, R^2^ values for the fixed effects (R^2^m) and entire model (R^2^c), REML criterion, and F values. Satterthwaite approximations were used to calculate denominator degrees of freedom.
(DOCX)

S4 Table Levin’s niche breadth species-pair comparisons. Species with significantly different LS mean values, based on the simplified model for Levin’s Niche Breadth. Estimate with standard errors, 95% confidence intervals, and p-values are provided for each species pair.
(DOCX)

S1 Fig Species accumulation curves. Species accumulation curves for each land-use category, with 95% confidence intervals, using 100 randomizations in EstimateS with bias correction.
(TIF)

S2 Fig Species richness estimation. Common richness estimators (ACE, ICE, Chao1, Jack1) based on number of individuals captured in each land-use category, calculated in EstimateS using 100 randomization.
(TIF)
