# Supplementary material for: Evidence of forest restoration success and the conservation value of community-owned forests in Southwest China using dung beetles as indicators
Source: PLoS One. 2018 Nov 8;13(11):e0204764. doi: 10.1371/journal.pone.0204764 (PMC6224038; doi:10.1371/journal.pone.0204764)
Supplement: S1 Table — Number of individuals of each species or morphospecies captured in each land-use category, and total species abundances and total abundances for each land-use category. (DOCX) [file pone.0204764.s001.docx]

|  | **Community Forest** | **Protected Forest** | **Restored Forest** | **Rubber Plantation** | **Total Abundance** |
| --- | --- | --- | --- | --- | --- |
| *Aphodius sp.* | 1 | 0 | 1 | 0 | 2 |
| *Catharsius granulatus* (Sharp, 1875) | 10 | 8 | 6 | 19 | 43 |
| *Copris sp.* | 10 | 4 | 4 | 1 | 19 |
| *Liatongus gagatinus* (Hope, 1831) | 5 | 2 | 6 | 17 | 30 |
| *Onthophagus anguliceps* (Boucomont, 1921) | 138 | 377 | 162 | 46 | 723 |
| *Onthophagus balthasari* (Vsetecka, 1939) | 0 | 1 | 9 | 0 | 10 |
| *Onthophagus dapcauensis* (Boucomont, 1921) | 1 | 3 | 4 | 0 | 8 |
| *Onthophagus diabolicus* (Harold, 1877) | 99 | 150 | 178 | 14 | 441 |
| *Onthophagus dissentaneus* (Balthasar, 1964) | 31 | 41 | 33 | 26 | 131 |
| *Onthophagus manipurensis* (Arrow, 1907) | 35 | 72 | 85 | 1 | 193 |
| *Onthophagus tricornis* (Wiedemann, 1823) | 107 | 109 | 62 | 68 | 346 |
| *Onthophagus zimmermaani* (Balthasar, 1959) | 23 | 17 | 76 | 9 | 125 |
| *Onthophagus sp1* | 385 | 227 | 170 | 4 | 786 |
| *Onthophagus sp2* | 0 | 5 | 2 | 0 | 7 |
| *Onthophagus sp3* | 174 | 299 | 165 | 38 | 676 |
| *Onthophagus sp4* | 14 | 10 | 37 | 1 | 62 |
| *Onthophagus sp5* | 0 | 1 | 0 | 0 | 1 |
| *Paragymnopleurus sp1* | 5 | 48 | 9 | 0 | 62 |
| *Paragymnopleurus sp2* | 2 | 0 | 0 | 4 | 6 |
| *Synapsis sp1* | 8 | 29 | 14 | 0 | 51 |
| *Synapsis sp2* | 8 | 4 | 5 | 2 | 19 |
| **Total Abundance** | **1056** | **1407** | **1028** | **250** | **3741** |

**S1 Table. Species abundances across land-use category.** Number of individuals of each species or morphospecies captured in each land-use category, and total species abundances and total abundances for each land-use category.
