# Supplementary material for: Evidence of forest restoration success and the conservation value of community-owned forests in Southwest China using dung beetles as indicators
Source: PLoS One. 2018 Nov 8;13(11):e0204764. doi: 10.1371/journal.pone.0204764 (PMC6224038; doi:10.1371/journal.pone.0204764)
Supplement: S2 Table — Number of individuals of each species or morphospecies on each bait type and total observed abundances and observed richness for each bait. (DOCX) [file pone.0204764.s002.docx]

|  | **Omnivore** | **Carrion** | **Elephant** |
| --- | --- | --- | --- |
| *Aphodius sp.* | 0 | 0 | 2 |
| *Catharius granulatus* | 29 | 11 | 3 |
| *Copris sp.* | 10 | 7 | 2 |
| *Liatongus gagatinus* | 2 | 28 | 0 |
| *Onthophagus anguliceps* | 497 | 210 | 16 |
| *Onthophagus balthasari* | 7 | 3 | 0 |
| *Onthophagus dapcauensis* | 8 | 0 | 0 |
| *Onthophagus diabolicus* | 218 | 217 | 6 |
| *Onthophagus dissentaneus* | 62 | 65 | 4 |
| *Onthophagus manipurensis* | 170 | 22 | 1 |
| *Onthophagus tricornis* | 104 | 105 | 137 |
| *Onthophagus zimmermaani* | 87 | 38 | 0 |
| *Onthophagus sp1* | 35 | 712 | 39 |
| *Onthophagus sp2* | 7 | 0 | 0 |
| *Onthophagus sp3* | 434 | 229 | 13 |
| *Onthophagus sp4* | 11 | 14 | 37 |
| *Onthophagus sp5* | 1 | 0 | 0 |
| *Paragymnopleurus sp1* | 49 | 12 | 1 |
| *Paragymnopleurus sp2* | 5 | 1 | 0 |
| *Synapsis sp1* | 35 | 15 | 1 |
| *Synapsis sp2* | 9 | 6 | 4 |
| **Total Abundance** | **1780** | **1695** | **266** |
| **Total Richness** | **20** | **17** | **14** |

**S2 Table. Species abundance across bait types**. Number of individuals of each species or morphospecies on each bait type and total observed abundances and observed richness for each bait.
