# Supplementary material for: Evidence of forest restoration success and the conservation value of community-owned forests in Southwest China using dung beetles as indicators
Source: PLoS One. 2018 Nov 8;13(11):e0204764. doi: 10.1371/journal.pone.0204764 (PMC6224038; doi:10.1371/journal.pone.0204764)
Supplement: S3 Table — Summary of the null model, global model, and final simplified models evaluating Levin’s Niche Breadth, with AIC, R2 values for the fixed effects (R2m) and entire model (R2c), REML criterion, and F values. Satterthwaite approximations were used to calculate denominator degrees of freedom. (DOCX) [file pone.0204764.s003.docx]

| **Model** | **AIC** | **R^2^m** | **R^2^c** | **REML** | **Co-variates** | **F-Value** | **DF (N,D)** |
| --- | --- | --- | --- | --- | --- | --- | --- |
| Null | 505.05 | 0.00 | 0.08 | 497.10 | -- | -- | -- |
| Global | 539.84 | 0.37 | 0.44 | 425.83 | Species | 8.25 | 20, 341.9 |
|  |  |  |  |  | Species’ Abundance | 21.95 | 1, 352.48 |
|  |  |  |  |  | Land-Use Category | 3.99 | 3, 33.86 |
|  |  |  |  |  | Total Abundance | 1.71 | 1, 1.81 |
|  |  |  |  |  | Rarefied Richness | 0.003 | 1, 29.89 |
|  |  |  |  |  | Species*Species’ Abundance | 1.91 | 18, 342.28 |
|  |  |  |  |  | Land-Use Category*Total Abundance | 1.20 | 3, 33.92 |
|  |  |  |  |  | Land Use Category*Rarefied Richness | 0.62 | 3, 27.36 |
|  |  |  |  |  | Land-Use Category*Species’ Abundance | 1.60 | 3, 351.19 |
| Simplified | 491.53 | 0.36 | 0.42 | 399.50 | Species | 1.43 | 20, 341.9 |
|  |  |  |  |  | Species’ Abundance | 4.69 | 1, 352.48 |
|  |  |  |  |  | Land-Use Category | 3.28 | 3, 33.86 |
|  |  |  |  |  | Species*Species’ Abundance | 1.88 | 18, 342.28 |

**S3 Table. Levin’s Niche Breadth model statistics.** Summary of the null model, global model, and final simplified models evaluating Levin’s Niche Breadth, with AIC, R^2^ values for the fixed effects (R^2^m) and entire model (R^2^c), REML criterion, and F values. Satterthwaite approximations were used to calculate denominator degrees of freedom.
