# Supplementary material for: Evidence of forest restoration success and the conservation value of community-owned forests in Southwest China using dung beetles as indicators
Source: PLoS One. 2018 Nov 8;13(11):e0204764. doi: 10.1371/journal.pone.0204764 (PMC6224038; doi:10.1371/journal.pone.0204764)
Supplement: S4 Table — Species with significantly different LS mean values, based on the simplified model for Levin’s Niche Breadth. Estimate with standard errors, 95% confidence intervals, and p-values are provided for each species pair. (DOCX) [file pone.0204764.s004.docx]

| **Species Pair** | **Estimate (SE)** | **95% CI** | **P-value** |
| --- | --- | --- | --- |
| *O. tricornis- Paragymnopleurus sp.1* | 0.3±0.13 | 0.069, 0.584 | 0.013 |
| *O. tricornis- C. granulatus* | 0.5±0.15 | 0.218, 0.822 | <0.001 |
| *O. tricornis- O. manipurensis* | 0.3±0.10 | 0.056, 0.450 | 0.012 |
| *O. tricornis- O. zimmermaani* | 0.3±0.11 | 0.070, 0.502 | 0.010 |
| *O. tricornis- O. dissentaneus* | 0.3±0.11 | 0.078, 0.501 | 0.007 |
| *O. tricornis- O. sp 1* | 0.4±0.11 | 0.217, 0.640 | <0.001 |
| *O. tricornis- O. sp 3* | 0.2±0.10 | 0.011, 0.405 | 0.039 |
| *O. tricornis- O. sp 4* | 0.5±0.13 | 0.237, 0.756 | <0.001 |
| *O. tricornis- Copris sp.* | 0.4±0.20 | 0.052, 0.832 | 0.026 |
| *Paragymnopleurus sp. 1- O. anguliceps* | -0.5±0.42 | -0.506, -0.007 | 0.044 |
| *C. granulatus- O. diabolicus* | -0.4±0.14 | -0.704, -0.137 | 0.004 |
| *C. granulatus- O. anguliceps* | -0.5±0.15 | -0.745, -0.156 | 0.003 |
| *C. granulatus- Synapsis sp. 2* | -0.7±0.29 | -1.243, -0.090 | 0.024 |
| *C. granulatus- O. sp3* | -0.3±0.15 | -0.603, -0.022 | 0.035 |
| *O. diabolicus- O. dissentaneus* | 0.2±0.09 | 0.005, 0.375 | 0.044 |
| *O. diabolicus- O. sp. 1* | 0.3±0.09 | 0.144, 0.513 | <0.001 |
| *O. diabolicus- O. sp. 4* | 0.4±0.12 | 0.158, 0.636 | 0.001 |
| *O. anguliceps- O. zimmermaani* | 0.2±0.11 | 0.009, 0.422 | 0.041 |
| *O. anguliceps- O. dissentaneus* | 0.2±0.10 | 0.018, 0.420 | 0.032 |
| *O. anguliceps- O. sp. 1* | 0.4±0.10 | 0.156, 0.561 | <0.001 |
| *O. anguliceps- O. sp. 4* | 0.4±0.13 | 0.174, 0.678 | <0.001 |
| *O. sp. 1-Syanspsis sp. 2* | -0.6±0.27 | -1.115, -0.085 | 0.037 |
| *O. sp. 1- O. sp. 3* | -0.2±0.10 | -0.415, -0.040 | 0.027 |
| *O. sp. 4- Synapsis sp. 2* | -0.6±0.28 | -1.200, -0.085 | 0.024 |
| *O. sp. 4- O. sp. 3* | -0.3±0.13 | -0.536, -0.040 | 0.023 |

**S4 Table. Levin’s Niche Breadth species-pair comparisons.** Species with significantly different LS mean values, based on the simplified model for Levin’s Niche Breadth. Estimate with standard errors, 95% confidence intervals, and p-values are provided for each species pair.
