# Supplementary figures and images for: Evidence of forest restoration success and the conservation value of community-owned forests in Southwest China using dung beetles as indicators
Source: PLoS One. 2018 Nov 8;13(11):e0204764. doi: 10.1371/journal.pone.0204764 (PMC6224038; doi:10.1371/journal.pone.0204764)

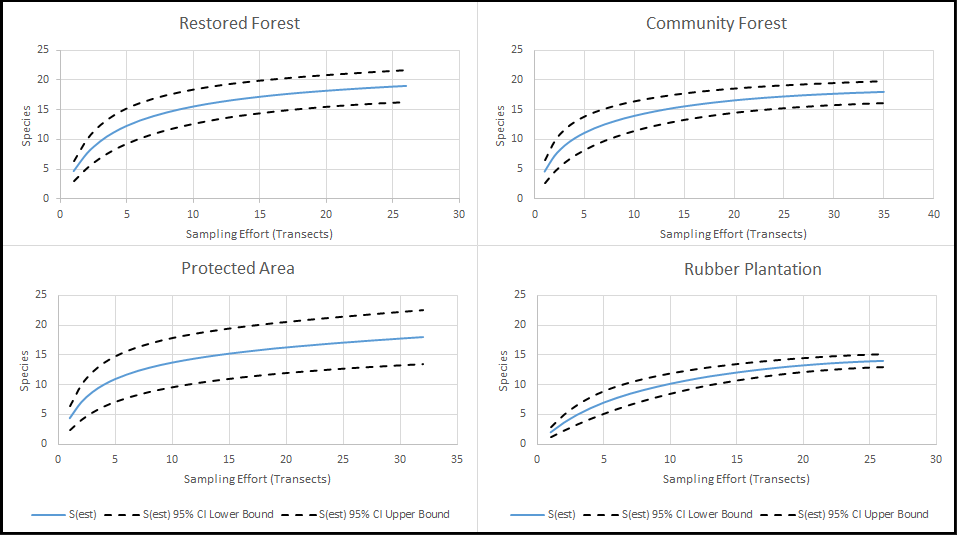

Supplement: S1 Fig — Species accumulation curves for each land-use category, with 95% confidence intervals, using 100 randomizations in EstimateS with bias correction. (TIF) [file pone.0204764.s005.tif]

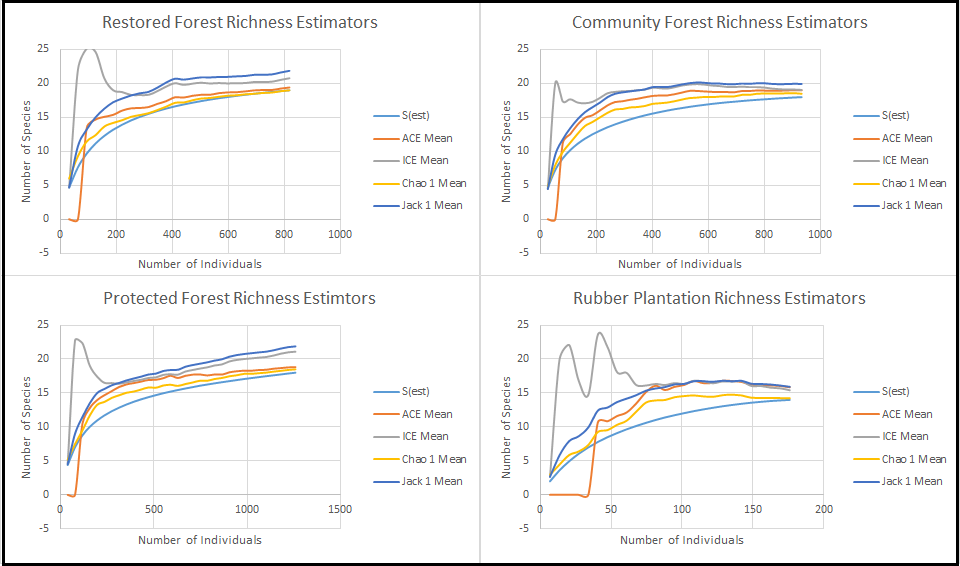

Supplement: S2 Fig — Common richness estimators (ACE, ICE, Chao1, Jack1) based on number of individuals captured in each land-use category, calculated in EstimateS using 100 randomization. (TIF) [file pone.0204764.s006.tif]
